# The Putative Antidepressant Mechanisms of Probiotic Bacteria: Relevant Genes and Proteins

**DOI:** 10.3390/nu13051591

**Published:** 2021-05-10

**Authors:** Elena Poluektova, Roman Yunes, Valery Danilenko

**Affiliations:** 1 Vavilov Institute of General Genetics, Russian Academy of Sciences, 117971 Moscow, Russia; epolu@vigg.ru (E.P.); valerid@vigg.ru (V.D.); 2 Faculty of Ecology, RUDN University, 117198 Moscow, Russia

**Keywords:** probiotics, psychobiotics, metabiotics (postbiotics), depression

## Abstract

Probiotic bacteria are widely accepted as therapeutic agents against inflammatory bowel diseases for their immunostimulating effects. In the last decade, more evidence has emerged supporting the positive effects of probiotics on the course of neurodegenerative and psychiatric diseases. This brief review summarizes the data from clinical studies of probiotics possessing antidepressant properties and focuses on the potential genes and proteins underlying these mechanisms. Data from small-sample placebo-controlled pilot studies indicate that certain strains of bacteria can significantly reduce the symptoms of depression, especially in depressed patients. Despite the disparity between studies attempting to pinpoint the bacterial putative genes and proteins accounting for these mechanisms, they ultimately show that bacteria are a potential source of metabiotics—microbial metabolites or structural components. Since the constituents of cells—namely, secreted proteins, peptides and cell wall components—are most likely to be entangled in the gut–brain axis, they can serve as starting point in the search for probiotics with concrete properties.

## 1. Introduction

In the last two decades, the field of human gut microbiota (HGM) has been marked by intensive and fruitful research. Today, the HGM is defined as a community of commensal, symbiotic and pathogenic microorganisms residing both inside the body and on its surface and that have adapted in the process of evolution to humans. The HGM consists of viruses, archaea, protozoa, fungi and bacteria, with the latter representing the largest and most studied group [1]. The HGM contains the most dense microbial population compared to other organs, which translates into high compositional and functional diversity [2]. Gut bacteria participate in the metabolic processes occurring in the intestines, such as the metabolism of carbohydrates and proteins, bile salts and polyphenols. They protect the human body from pathogens and maintain the integrity of the intestinal barrier. Finally, they synthesize hundreds of biologically active substances, such as lactic and acetic acids, hydrogen peroxide, bacteriocins, short-chain fatty acids, vitamins, proteins, peptides, cell wall components and neuroactive compounds, all of which shape and ensure the normal functioning of the immune, nervous and endocrine systems [2,3,4]. Of particular interest are the immunomodulatory properties of CpG motifs, as seen in bifidobacteria characterized by notoriously high GC content genomes. CpG motifs—stretches of DNA rich in their contents of the deoxyoligonucleotides cytosine and guanine—were shown to activate both the innate and adaptive immune responses via Toll-like receptor 9 (TLR-9) [4]. Today, the microbial composition of the human gut is an important health indicator. Many studies have found correlations between the HGM composition and human diseases (obesity, hypertension, cardiovascular disease, diabetes, cancer, inflammatory bowel disease (IBD), gout, depression and arthritis). The HGM also correlates with overall infant health and longevity [5].

As our knowledge of the composition and function of the HGM has expanded, it has become clear that certain health-conferring bacteria can be put to use for therapeutic purposes. Probiotics are defined as live microorganisms that, when administered in adequate amounts, exert beneficial effects on the host organism. Since the observation made by the Russian scientist Elie Metchnikoff regarding the positive influence of lactobacilli on health [6], probiotic properties have been uncovered in many microbial genera and species: *Bifidobacterium*, *Streptococcus*, *Enterococcus*, *Lactococcus*, *Bacillus*, *Escherichia coli* M-17, *Saccharomyces boulardii* and others. Probiotics are especially effective in treating chronic inflammatory bowel diseases and the metabolic syndrome while virtually showing no side effects [7]. The prospect of using certain probiotics as an alternative treatment for depression was delineated many years before the term psychobiotics was coined [8,9]. Numerous studies have followed and further strengthened the concept of psychobiotics by demonstrating the salubrious effects of bacteria-based therapy on neurodegenerative and psychiatric diseases such as depression, schizophrenia, epilepsy, cerebral ischemia, Parkinson’s disease, Alzheimer’s disease, insomnia, autism spectrum disorder and others. Nevertheless, the bulk of the research in this field has been carried out on animal models, mimicking, to a certain extent, the symptoms of these diseases. Moreover, the majority of studies were not followed up in humans; among the strains that were tested in clinical settings, few were proven effective [10,11,12].

Depression persists in today’s world as the most common psychiatric disorder. The numbers we have today remain a rough estimate. According to the World Health Organization, some 300 million suffer from depression; the actual number is likely higher than that [13]. Depression qualifies as a socially significant disease, since it significantly reduces the quality of life of patients. Depression is no less a burden for patients than it is a burden for their families. Some of the major symptoms of depression include a lingering bad mood, anhedonia, fatigue, lethargy and anxiety and, if left untreated, can become a high risk factor for suicide. The current nascent paradigm stipulates that depression is accompanied by chronic subclinical inflammation that stems from an aberrant gut microbiota, increased permeability of the gut barrier and impaired immune status [14]. There is sufficient evidence that bacterial metabolites are implicated in the pathogenesis of depression [15]. Since antidepressants are effective only in a subset of patients and are also plagued by their infamous side effects—insomnia, fatigue, anxiety, confusion, gastrointestinal symptoms, dry mouth, skin redness, itch and photophobia—there is an ever-increasing demand for safer drugs, which could be satisfied by probiotics [16].

## 2. Selection of Potential Antidepressants among Bacteria

The first step in selecting for psychobiotic strains is to conduct a wide-scale in vitro assessment of the general probiotic properties of bacteria. Bacterial strains should be selected based on their ability to withstand the lethally acidic environment of the stomach and the bile salts in the duodenum. At this stage, the strain’s ability to adhere to intestinal epithelial cells and to suppress the growth of pathogenic bacteria should be investigated as well. Then, preliminary safety studies are needed to confirm the absence of mobile genes, accounting for transferable pathogenicity and antibiotic resistance. The next step is to look for other properties, such as the production of active compounds—namely, bacteriocins, organic acids and exopolysaccharides—and the conversion of carbohydrates, proteins and other food components into short-chain fatty acids and other molecules. Expanding our knowledge of the technical characteristics of potential psychobiotics is crucial for production. The viability of the strains in the end product depends on their ability to withstand the freeze-drying process. Just as important is to test the viability of the strains during long storage. There are tests specifically designed to check the activity of the strain at all stages of the manufacturing process. After all these steps are completed, the strain’s specific activity, whether antidepressant, anti-inflammatory, anticarcinogenic or immunomodulatory, should be studied in both in vitro and in vivo systems. Finally, perhaps the most necessary and revealing part of this protocol is to validate the strain’s effects in human volunteers [17].

The antidepressant potential of bacteria has been attested to by hundreds of studies conducted on laboratory animals—mainly rats and mice. The bacterial strains tested in these studies belonged predominantly to the genera *Lactobacillus* and *Bifidobacterium* [18]. These studies also emphasized the strain-specific rather than species-specific nature of these properties [19]. Most experiments are designed similarly, and they involve the administration of a single or a mixture of strains of bacteria to a control group made up of conventional or gnotobiotic animals as opposed to a control group receiving a placebo. At the same time, the animals are exposed to a stressor for the duration of the experiment and tested afterwards in relevant behavioral tests, such as sucrose preference, forced swimming test, elevated plus maze, open field and others. The biochemical results show that specific probiotic strains can impact the HPA (hypothalamic–pituitary–adrenal) axis by lowering the corticosterone and ACTH (adrenocorticotropic hormone) blood levels. The levels of dopamine, serotonin, tryptophan and other neurotransmitters are also normalized in various regions of the brain and the blood serum. Other effects involve the regulation of the activity of gamma-aminobutyric acid (GABA) receptors in various regions of the brain and the level of the brain-derived neurotrophic factor in the hippocampus and blood serum. Most importantly, some probiotic bacteria can reduce inflammation by inhibiting the release of a C-reactive protein and other proinflammatory cytokines (IL-6, IFN-γ, TNF-α and IL-1β) and upregulating anti-inflammatory cytokines such as IL-10 in the blood plasma and the spleen. A recent in vitro study conducted by Dyakov et al. demonstrated that the protein FN3 produced by bifidobacteria possesses TNF-α-binding properties. *Bifidobacterium longum* contains the gene cluster PFNA, which is presumably involved in species-specific communication between the bacteria and their hosts. The gene cluster PFNA consists of five genes, including *fn3*, which codes for a protein containing two fibronectin type III domains. *E. coli* BL21 (DE3), containing the recombinant plasmid pET16b:fn3, was genetically engineered to produce a recombinant FN3 protein [20]. Probiotic bacteria are also known for their ability to activate many antioxidant enzymes in the brain and blood serum—namely, superoxide dismutase and glutathione peroxidase. The relationship between the gut microbiome-derived enzymes and the human organism remains largely uncharted territory. Enzyme preparations, for instance, work differently in the human body. The most important question is how enzymes/metabiotics penetrate human cells. These could be type III secretion systems, bacteriophage infections, Gram-positive bacterial extracellular vesicles, ergothioneine and microbe-derived metabolites such as *L. reuteri* [21,22,23,24]. Unfortunately, none of the abovementioned mechanisms were described in other species. Probiotics can interact with the cell components; it is also plausible that probiotics interact with the metabolites of human cells, thus giving rise to new compounds. However, these are often contradictive and speculative pathways that will not be addressed in this study. Probiotics can achieve this in many ways, including by the direct production of antioxidant enzymes or by reduction of the lipid peroxidation levels in the blood [12,25]. Aside from that, the role of probiotic bacteria is central to sustaining the integrity of the gut–brain barrier and the balance of the gut microbiota composition [18].

## 3. Clinical Studies of Putative Psychobiotics

Since animal studies a priori do not guarantee the reproducibility of their results in humans [26], it is crucially important to study psychobiotics in humans. Fortunately, several dozen studies corroborate the positive effects of probiotics on the symptoms of depression, anxiety and stress in randomized, double-blind, placebo-controlled studies. The maximum number of participants in these studies amounted to 423 people [27]. On average, the number of participants in each study ranged between 30 and 100. The design differs significantly among studies: some tested putative psychobiotics on healthy volunteers, while others subjected volunteers to stress simultaneously or recruited patients with depression/anxiety. Some of the species commonly used are *L. helveticus*, *L. rhamnosus*, *L. casei*, *L. gasseri*, *L. plantarum*, *L. acidophilus*, *L. delbrueckii* subsp. *bulgaricus*, *B. longum*, *B. longum infantis*, *B. breve*, *B. bifidum*, *B. animalis* subsp. *lactis*, *Bacillus coagulans*, *Clostridium butiricum* and *Streptococcus thermophilus*. Typically, both approaches involving the administration of a single strain or a mixture of strains are equally effective [28,29,30,31,32,33,34,35,36,37,38,39,40,41,42,43]. Some probiotics are mixed with pharmaceutical aids, such as maltodextrin and starch, or prebiotics such as oligosaccharides and inulin, which, in the latter cases, are qualified as synbiotics. Prebiotics are substrates that are selectively used by the host microorganism to confer a specific health benefit (the International Scientific Association for Probiotics and Prebiotics—ISAPP). Synbiotics refer to food ingredients or dietary supplements combining probiotics and prebiotics and producing a form of synergism—hence, synbiotics [44].

In addition, synbiotics themselves possess antidepressant properties and can complement the effects of psychobiotics [42]. Commonly, a daily dose of 10^9^–10^10^ microbials cells is administered for a duration of 3 to 24 weeks. The mode of delivery can take the form of a capsule or a yogurt [38]. Despite all the accomplished milestones in this field, the specific role of the different constituents of probiotics remains understudied.

Not all psychobiotics were created equal: some strains were shown to be effective in attenuating the symptoms of depression but not those of anxiety [31,32,42], while others were suited for treating anxiety but not depression [28]. There is also a third group of psychobiotics that counter the symptoms of both depression and anxiety alike [30,34,38,43]. However, there are a few issues with psychobiotics that have yet to be solved. As with other treatments, not all subjects are responsive to psychobiotics. The outcome measures used for the assessment of the magnitude of anxiety and depression vary widely across studies. Even within the same study, different questionnaires do not always yield the same results [30]. Psychobiotic research has extended beyond behavioral testing and attempts to pinpoint the concrete mechanisms at work. Overall, these include gut microbiota modulation [28,30], keeping the cortisol levels in the blood plasma and saliva in check [29,30,35,36,43] and anti-inflammatory and antioxidative actions [33,35,39]. Some of the more relevant changes are increases in the BDNF levels in the blood serum [43] and in the neural activity of the prefrontal cortex [29], enhancement of the serotonin pathway [35] and other changes in the brain regions affected during depression [31]. In addition, many of these stains demonstrated immunomodulatory and anti-inflammatory properties in upper respiratory and gastrointestinal infections. These results further attest to the fact that psychobiotics are not restricted to concrete taxonomic groups. Psychobiotics can be of different species and genera, as long as they are suited for treating nervous system diseases.

Consistency in the exerted effects is another important aspect of psychobiotic research [28,45]. Most importantly, psychobiotics were especially helpful when consumed by patients with proven clinical depression [32,33,39,40,41,43], albeit nonresponsive to antidepressant therapy [34]. This interesting and crucial aspect of probiotics pervades most of psychobiotics research on depressed patients and thus justifies the search for new psychobiotics and their use in clinical practice. Gender-specific and age-related different effects are also common [31]. Moreover, following the cessation of intake, the achieved therapeutic progress lingered for several weeks, whereas in other cases vanished shortly after [29]. The effects of psychobiotics are far from uniform. The effects of some of them can last longer than others. All this points to the differences between psychobiotics, which should be taken into account in the process of drug development. It is noteworthy that none of the studies reported serious side effects of probiotics.

The unique characteristics of psychobiotics are not restricted to a single taxonomic group but, rather, have been identified by unrelated authors in different species throughout bacterial families. This makes it difficult to draw parallels between different strains and derive the appropriate conclusions. We believe that the properties of the probiotics (psychobiotics) are not determined by any single gene or enzyme but by a set of properties. Therefore, at the current technological level of development of microbiology, we cannot single out these properties and study them in laboratories.

Today, Cerebiome^®^—the probiotic formulation manufactured by the Canadian company Lallemand Health Solutions, is one of the most studied psychobiotics in this field. The composition consists of two strains: *L. helveticus* R0052 and *B. longum* R0175. The combination of these two strains has proven effective in reducing the symptoms of depression [36,40,46], despite some authors failing to confirm their antidepressant action [47]. The reason possibly lies in the differences between the participants of these studies. Yet, these results are to be regarded as preliminary, since they were not replicated in large enough samples. Other commercial products based on the strains *Lactobacillus rhamnosus* JB-1, *Lactobacillus helveticus* NS8 and other bacterial species *Mycobacterium vaccae* and *Bifidobacteria infantis* were less successful than the psychobiotics, despite their antidepressant and immunomodulatory properties.

Many independent meta-analyses of randomized, double-blind, placebo-controlled trials that have been conducted over the last two years have reached overlapping conclusions [48,49,50,51,52,53]. Probiotics exhibit statistically significant positive effects on the symptoms of depression in humans compared to placebos. These studies also suggest that the action of probiotics is highly dependent on both the probiotic strain and the target host. For instance, in the study of Amirani et al., the authors summarized that the use of probiotics significantly alleviated the symptoms of patients when rated on the Hamilton Rating Scale for Depression (HDRS) but not on the Beck Depression Inventory (BDI) [52]. Many factors contribute to whether probiotics will exert any effects at all. The presence of tangible symptoms, comorbidities, adjuvant therapy with prebiotics or antidepressants, gender and age of the subjects are all considered important variables. As shown in the mentioned studies, probiotics are more effective in patients with depression and anxiety than people exposed to stressful events [50]. This is the most important merit of probiotics. These data may be indirect evidence of the different underlying causes of major depressive disorder (MDD) and other types of depression. Another important conclusion is that, in order for probiotic therapy to be effective, it needs to be long-lasting [49]. All studies also agree that, despite the evidence in favor of the antidepressant potential of probiotics in humans, the results are still preliminary. The effectiveness of probiotics needs to be studied in sufficiently large cohorts, allowing to determine the optimal doses and regimens, as well as the duration of the obtained effects. Only then probiotics might be acknowledged as effective antidepressant therapy.

## 4. Active Metabolites of Probiotic Bacteria and Genes Involved in Their Synthesis

The most pressing issue in probiotic research is the identification and isolation of metabolites, accounting for their effects. Needless to say, the identification of metabolites is most important for understanding how probiotics work. Once accomplished, it will uncover the sought-after knowledge required for a mechanistic approach towards the development of probiotics. Bacterial metabolites, which encompass metabiotics (postbiotics), structural components, signaling molecules of probiotic bacteria with a defined chemical structures and others, are potent modulators of a host’s physiological functions [54,55,56,57]. The research aimed at identifying metabiotics is usually conducted in animal models.

Probiotic bacteria are known to synthesize a wide range of neuroactive compounds, potentially being involved in the communication with their host: GABA, serotonin, dopamine, adrenaline, norepinephrine, histamine and others [58,59,60,61]. Gut bacteria possess various machinery for GABA production. The most common pathway of GABA production in bacteria occurs via the decarboxylation of glutamate (*gad* ABC genes); the less common pathways are those of putrescine metabolism (genes *puu* ABCD and *ygj*G, *ydc*W) [62]. Only a few studies have shown that microbe-derived GABA alleviates the symptoms of depression and anxiety in laboratory animals [63,64,65].

In the study of Marin et al., which was carried out on mice, the antidepressant effect of *L. reuteri* was presumably related to the production of hydrogen peroxide (H_2_O_2_) and to the strain’s inhibitory effect on indoleamine-pyrrole-2,3-dioxygenase (IDOI) [66]. Hydrogen peroxide is formed inside cells of lactobacilli following NADH or pyruvate oxidation and decomposition of the reactive oxygen species. Hydrogen peroxide participates in redox signaling, and it is oxidized by peroxidases [67]. Although hydrogen peroxide is present in the majority of lactobacilli, normally, it does not produce antidepressant effects. The levels of H_2_O_2_ production in *L. reuteri* is probably determined by regulatory mechanisms.

Another active enzyme is carboxyesterase, which is produced by *B. breve* ATCC 15700 (WP_003829800.1, WP_003829196.1, WP_003828396.1 and WP_003828023.1). Carboxyesterase converts the antidepressant albiflorin (C_23_H_28_O_11_), a plant-derived metabolite possessing neuroprotective properties, into benzoic acid. The latter is capable of penetrating the brain through the blood–brain barrier [68]. Once inside the brain, benzoic acid inhibits D-amino acid oxidase (DAAO), thereby exhibiting the antidepressant effect. However, benzoic acid is used as a medication for schizophrenia, not depression [68].

Gut bacteria break down carbohydrates, otherwise indigestible in humans (oligosaccharides, inulin and human milk oligosaccharides) [69], into short-chain fatty acids (SCFA)—namely butyric, acetic and propionic acids. SCFAs reach their highest concentration in the colon, where they can be either used by enterocytes as a source of energy or transported into the blood circulation. Many studies refer to the major regulatory role of SCFAs, involving them in a wide range of diseases. Overall, they are considered indispensable for immune homeostasis of the gut [70]. When mice were exposed to chronic stress while receiving the probiotic strain *L. plantarum* MTCC 9510, they showed a decrease in depressive-like behavior accompanied by an increase in the amount of fecal SCFAs levels [71]. The administration of *Faecalibacterium prausnitzii* ATCC 27766 to stressed rats also conferred preventive and therapeutic effects, attested by behavioral tests, while increasing SCFA caecal contents [72]. In a mouse model of vascular dementia, the ingestion of *Clostridium butyricum* CGMCC 9831 reduced the cognitive and histological indicators and restored the butyric acid normal levels in both the feces and the brain [73]. However, more studies are needed to ascertain whether the mounting SCFA levels are a direct consequence of the activity of these bacteria or whether probiotics affect SCFA levels by merely stimulating other SCFA-producing bacteria. The key enzymes involved in the different pathways of butyrate production are butyrate kinase and butyryl-CoA dehydrogenase [74]. As for propionate production, the main enzymes are lactyl-CoA dehydratase, propionaldehyde dehydrogenase and methylmalonyl-CoA decarboxylase [75].

As noted above, depression is associated with low-grade inflammation, and most probiotics showing antidepressant results possess immunomodulatory properties. It is therefore feasible that bacteria, which elicit an immunomodulating capacity, might as well exert the effects of antidepressants, although that link is yet to be confirmed. The active bacterial metabolites involved in immunomodulation have been studied exhaustively [3]. These include secreted proteins and peptides.

Seven active peptides were derived from a 15-kDa protein produced by *Faecalibacterium prausnitzii* [76]. The seven ions/peptides were initially identified using MALDI-TOF in the supernatant of *F. prausnitzii* and shown, each in isolation, to exert inhibitory effects on the secretion of proinflammatory cytokines by intestinal epithelial cells. Later, an in silico analysis confirmed that all seven peptides were part of a 15-kDa protein called MAM (microbial anti-inflammatory molecule or named after its discoverer Marie-Anne Maubert). The transfection of epithelial cells with a 15-kDa cDNA fragment led to a significant decrease in the activation of the NF-κB pathway, with a dose-dependent effect. Finally, the use of a food-grade bacterium, *Lactococcus lactis*, delivering a plasmid encoding this protein was able to prevent colitis in mice.

The serine-threonine peptide STp is a fragment of a protein secreted by *L. plantarum* BMCM12 when exposed to intestinal proteases [77]. The treatment with STp partially restored the normal functioning of colonic dendritic cells affected by ulcerative colitis. Bacterial-derived metabolites such as STp might lead the way to a new era of probiotic products, laying the foundation for the nondrug dietary therapy of inflammatory bowel disease.

The secreted proteins have been reported in *L. rhamnosus* and *L. casei* strains, such as p75 and p40, and cell wall peptidases containing CHAP and NlpC/p60 domains (*LCABL_00230* and *LCABL_02770* found in *L. casei* BL23) [78,79]. These proteins, in a purified form, activated protein kinase B, inhibited apoptosis and stimulated the growth of human and mouse cells lines of colonocytes. p75 and p40 were especially potent protective agents against TNF-induced epithelial damage in the colon. These results bolster support for the potential of bacterial components for treating cytokine-mediated gastrointestinal disorders.

Secreted antigen A (SagA) belongs to the NlpC/p60 family of peptidoglycan hydrolases reported in *Enterococcus faecium* Com15, hydrolyzing Lys-type peptidoglycan fragments [80,81]. SagA protected *Caenorhabditis elegans* against *Salmonella* pathogenesis by increasing its tolerance to infection.

The secreted protein product of the gene *HMPREF0539_2242* was reported in *L. rhamnosus* LMS2-1; so far, no structural domains have been published [82].

A 32-kDa protein encoded by the gene *Amuc_1100* was found in *Akkermansia muciniphila*; the protein is part of the outer membrane, and it is stable at pasteurization temperatures [83]. *Amuc_1100* improves the gut barrier function and has been shown to be safe in humans. It was projected that *Amuc_1100* could be used as a treatment for obesity and associated disorders.

Lactosepin, a protein secreted by *L. paracasei* LMG S-29188, one of the strains of the probiotic preparation VSL #3, served the function of a cell wall protease. A human -derived isolate of the species *Lactobacillus casei* was shown to encode lactocepin and degrade IP-10, a lymphocyte-recruiting chemokine. A follow-up study demonstrated in a model of murine colitis that the disruption of the gene *prtP* encoding lactocepin tuned down the *L. casei* ability to reduce the IP-10 levels and inflammation in the cecum. These results imply the future use of lactosepin in IBD therapy [84].

Serpin is a serine protease inhibitor produced by *B. longum*, *B. breve* and *B. dentium* [85]. In the strain *B. longum* NCC2705, the protein is encoded by the gene *BL0108* [86]. It was suggested that serpins participate in maintaining the barrier function and inhibit the action of elastases.

Unfortunately, unlike the compounds mentioned further down, the antidepressant properties of these proteins and peptides were not tested.

Bacterial strains are used in probiotics produce a number of neurotransmitters identical to those secreted in the human body. In addition, some of these molecules were shown to possess immunomodulatory properties—namely, *L. brevis* BGZLS10-17-derived GABA and *L. reuteri*-derived histamine [87,88]. Histamine is a key biogenic amine that is implicated in protein kinase C signaling in mammalian cells. Apart from being an important mediator of allergic inflammation, histamine’s effects extend to the central nervous system, especially to certain areas such as the hippocampus. Microbes can also synthesize histamine to maintain the intracellular pH. Bacteria form histamine from its precursor histidine using histidine decarboxylase. In *L. reuteri*, the key gene involved in this pathway is *hdcA* (*HMPREF0535_RS02485* in *L. reuteri* MM2-3 and *HMPREF0536_11827* in *L. reuteri* MM4-1A). Histamine activates the H2 and H1 histamine receptors in humans. However, the activation of the latter does not trigger an inflammatory response due to another molecule produced by *L. reuteri* simultaneously. Diacylglycerol kinase inhibits the activation of the H1 histamine receptor by metabolizing its substrate [89].

A stark example of depressogenic cell wall components or outer cell membranes is that of lipopolysaccharides (LPS), which are expressed by Gram-negative bacteria. LPS acts as an endotoxin, because it binds the CD14/TLR4/MD2 receptor complex in many cell types but especially in monocytes, dendritic cells, macrophages and B cells, which promotes the secretion of proinflammatory cytokines, nitric oxide and eicosanoids. In humans and rodents, the systemic administration of LPS causes a spectrum of behavioral alterations that resemble depression, including depressed mood, fatigue and psychomotor retardation in humans, as well as anhedonia, decreased activity, cognitive dysfunction and altered sleep in rodents [90].

In Gram-positive bacteria, the cell wall components are mainly known for their immunomodulatory properties. No studies have reported direct antidepressant effects so far. The cell wall components of bacteria are important immunomodulatory biological agents. A few examples of the cell wall components are exopolysaccharides (EPS), teichoic acids and lipoproteins, usually making up Gram-positive bacteria, which constitute the majority of those used in probiotics. EPS are high molecular weight water-soluble linear or branched polymers (dextran, xanthan, alginate, gellan and pullulan) composed of repeating mono- or oligosaccharides. The immunomodulatory, anticancer, antioxidant, antiulcer, antibiofilm and blood glucose cholesterol-lowering properties and antihypertensive activity EPS have been demonstrated in many studies [91,92]. The activity of EPS depends on their composition due to the differences in their physical and chemical characteristics [93]. Considerable immunomodulatory activity was attributed only to certain EPS [94,95]. EPS synthesis is encoded by many genes and usually organized into operons [96].

The most characterized teichoic acid in terms of immunomodulatory activity is lipoteichoic acid (LTA). LTA was studied in *L. plantarum*, *L. paracasei*, *L. gasseri* and others [97,98]. Lipopeptides (Lpp), being the main Toll-like receptor 2 agonists produced by Gram-positive bacteria, also exhibit immunomodulatory activity. Toll-like receptors are expressed by the host; bacteria do not express them. The number of genes involved in the synthesis of lipoproteins varies from species to species and makes up a significant part of the bacterial genomes, which are 1–3% of total genes of any given strain [99]. An important protein of this category is the lipid-binding prolipoprotein diacylglycerol transferase (encoded by the gene *lgt* and annotated as *lp_0755* in *L. plantarum* WCFS1) [100]. Components of the bacterial cell wall can retain their immunomodulatory activity, even after heat inactivation [97].

The immunomodulatory activity of bacteria could be explained by their nucleic acids. For instance, unmethylated CpG motifs of *B. longum* NCC2705, *B. adolescentis* ATCC15703 and *B. longum* subsp. *infantis* ATCC 15697 are capable of binding to Toll-like receptor 9 [4,101]. This effect was similarly observed in oligonucleotides of *L. casei* ATCC 27092—namely, TTTTGCCG and the total RNA and genomic DNA of phagocytosed *L. gasseri* OLL2809 cells [102,103].

Moreover, immunomodulatory activity is not limited to individual metabolites and cell components of probiotic bacteria but also extends to extracellular vesicles—secreted nano-sized molecules and fragments of structural components of bacterial cells [104,105,106,107]. To cite one example, the extracellular vesicles of *L. plantarum* WCFS1 enhanced the immune response of enterococci-infected nematodes and human cell lines [108]. A recent preliminary study confirmed the relation between microbial-derived extracellular vesicles and depression. In the study, the extracellular vesicles derived from *Lactobacillus plantarum* were shown to increase the BDNF expression in cultured hippocampal neurons and produce antidepressant-like effects in mice [109].

The data on the active components of potential probiotic bacteria are inconclusive and often fragmented. Nevertheless, they suggest that the active components of bacterial cells constitute a large and diverse pool with secreted proteins/peptides and cell wall components at its center. During the past two years, many review papers have discussed the immunological and antioxidant potential of postbiotics, mainly derived from bacterial cell components, which can be utilized for the production of new-generation antidepressants [55,56,57]. Unfortunately, the microbial compounds and metabolites (postbiotics) were only shown to have an impact on the indicators that are indirectly linked to depression, such as the anti-inflammatory, antioxidant and antimicrobial effects. Thus, the potential of postbiotics in treating depression can only be inferred from what we know about their role in immunomodulation [110].

## 5. Conclusions

Small-sample placebo-controlled clinical trials have repeatedly shown that probiotics have proven effective in countering the symptoms of depression. Unfortunately, all the studies addressing the antidepressant effects of probiotics are not sufficiently large in number. Meta-analyses need to be applied to the large-scale studies. The antidepressant activity of probiotics is restricted to particular strains belonging, in most cases, to either of the genera *Lactobacillus* and *Bifidobacterium*. Probiotics exert distinct effects on the host organism that depend on the strain used and the targeted cohort. These effects were most salient in people diagnosed with depression. Conversely, it is important to keep in mind that these are preliminary data that require validation in sufficiently large samples. One of the consequences of the lack of adequate trials is that registered drugs of this type remain absent from the market altogether.

Another relevant issue is that research addressing the active components of probiotics, including those conferring antidepressant effects, is relatively scarce. Contrastingly, the data on bacterial components that exhibit immunomodulatory properties, although they have been studied more thoroughly, remain inconclusive and fragmentary. Overall, the major active components can be classified as secreted proteins/peptides and cell wall components. The diversity of active metabolites, their targets and mechanisms of action, highlight the great potential of metabiotics as drugs for the prevention and treatment of depression. The same data imply that the mode of action of probiotics is determined not just by one molecule but, rather, by the totality of metabolites secreted by a strain. The knowledge of proteins and genes involved in the production of active metabolites can be utilized to search for bacterial strains with concrete properties. A few examples of such a successful selection of strains have been reported. The consensus today on this issue is that a line should be drawn between orally administered GABA and microbial-derived GABA, which is not the same thing. The impact of GABA on the nervous system appears to be indirect—via the enteric nervous system—which allows to circumvent the question of GABA’s inability to cross the blood–brain barrier. Another possible link between microbial-derived GABA and depression is via the modulation of the gut microbiota. This is supported by a recent discovery showing that there are bacteria that consume GABA and use it as their sole source of energy [111]. For instance, the *L. plantarum* and *B. adolescentis* strains, selected based on their ability to produce GABA, have been shown to reduce the depressive-like behavior in mice [63].

On a final note, the COVID-19 pandemic poses a great threat to public health, mainly due to a panoply of troubling side effects observed in millions of people recovering from the disease. Depression, in particular, has been on the rise since the beginning of the pandemic [112,113]. Thus, as more people with disrupted gut microbiomes are affected by COVID-19, there is a desperate need for adequate rehabilitation programs that would be significantly improved if new antidepressants such as those based on probiotics and postbiotics were accessible.

## Data Availability

Not applicable.
